# Amethopterin: A Toxic Tumour Growth Inhibitor

**DOI:** 10.1038/bjc.1952.42

**Published:** 1952-12

**Authors:** J. G. Bennette


### AMETHOPTERIN: A TOXIC TUMOUR GROWTH INHIBITOR.

J. G. BENNETTE.

*From the Courtauld Institute of Biochemistry, Middlesex Hospital, London, W.1.*

Received for publication October 14, 1952.

THE introduction of the toxic analogues of pteroylglutamic acid in the treatment of human leukaemias reported first by Farber, Diamond, Mercer, Sylvester and Wolff (1948), and subsequently by many other workers, has led to an extensive investigation of the properties of these compounds. The rather scanty information which was at first available to afford theoretical support for the rationale of this treatment has become expanded into a literature of considerable proportion. Burchenal and his associates (1951) and Petering (1952) have reviewed the more important investigations into the properties of pteroylglutamic acid analogues and quote references to the work which has substantially reduced the empiricism of this chemotherapeutic approach. Although much enthusiasm has been developed for the antineoplastic potentialities of these compounds, their usefulness in treatment has been greatly limited by their extreme toxicity, perhaps the most striking of their properties. Their application is being increasingly restricted on this account.

The very poisonous character of these drugs has received much detailed consideration in the clinical context, but in common with other toxic drugs used in this field, less theoretical attention has been directed towards the problems connected with the assessment of their usefulness than would seem to be justified in view of the importance of this aspect of chemotherapy. In the great bulk of experimental work, 4-amino-$N_{10}$ methyl pteroylglutamic acid (amethopterin) and 4-amino pteroylglutamic acid (aminopterin) have been used. The former, being some ten times less toxic than aminopterin (Ferguson, Thiersch and Philips, 1950), has been used in the work reported here.

Sugiura, Moore and Stock (1949) reported that aminopterin produced growth retardation of a transplanted sarcoma, an adenocarcinoma and a melanoma in mice, and noted that inhibition only occurred at toxic levels of dosage. It was suggested, nevertheless, that this association between the tumour retarding effect and the toxicity was compatible with the view that aminopterin had a definite effect against tumour tissue. Schoenbach, Goldin, Goldberg and Ortega (1949) have also reported growth regressions of Sarcoma 180 in response to aminopterin and observed that body weight losses of up to 20 per cent were sustained by the mice, but concluded from the greater percentage loss sustained by the tumours that the drug was acting selectively. Moore, Stock, Sugiura and Rhoads (1949) obtained inhibition of the growth of Sarcoma 180 using amethopterin and noted the association between toxicity and tumour growth retardation. The importance of this was not believed to be great enough to modify the conclusion that the compound was a powerful inhibitor of the growth of this tumour. The evidence presented here, though necessarily circumstantial, supports the contention that most of the anti-neoplastic activity of amethopterin for Sarcoma 180 and Sarcoma

37 is the result of an unselective toxic state induced as a result of treatment in the animals carrying the tumours. The sex difference in toxicity reported by Goldin, Greenspan, Goldberg and Schoenbach (1950) was confirmed and it was noted in preliminary experiments that tumour growth retardation was obtained only in tumours carried by the male mice which also showed a lower tolerance to the drug. It was decided to investigate this sex difference in order to discover whether the difference in the growth of the tumours between the sexes was a function of the sex of the host acting by enhancing the effect of the drug on the tumour or whether it was due to a secondary effect caused only by the different susceptibility to intoxication.

### MATERIALS AND METHODS.

The mice used throughout were mixed strain albinos obtained from reliable dealers and used when they had attained a body weight of between 25 and 30 g. They were housed in an air-conditioned atmosphere, the temperature of which was maintained at 24 $\pm$ 4° C. Wire cages of a size comfortably to accommodate 20 mice were fitted with wire floors carrying bent wire feet so that the cages were raised about 2 cm. above the level of the metal trays in which they were placed. Each tray was lined with a sheet of absorbent paper. Water was supplied *ad lib.* from bottles fitted with long stems which reached to within 2 cm. of the floors of the cages. The rat cake used had the following composition :

| | | |
|---|---|---|
| Moisture | 14·3 | per cent. |
| Soluble carbohydrate | 53·4 | ,, |
| Protein | 20·0 | ,, |
| Fat | 3·8 | ,, |
| Fibre | 3·3 | ,, |
| Ash | 5·2 | ,, |

It was finely ground in a mill and dried at 37° C. for 24 hours in a thin layer. The degree of desiccation obtained in this way was found to be such that there was a negligible alteration in the water content of the ground food when this was exposed to the conditions existing in a cage of mice for 24 hours. A weighed excess of the food was given to the mice in a suitable arrangement of 2 oz. and 4 oz. glass ointment jars fitted with metal screw caps in each of which a circular hole, 3 cm. in diameter, had been cut to allow access to the food. On the surface of the food was placed a wire grille of such a size that it easily fell in the jar as the food was eaten, but could not be pulled through the hole in the screw cap. This arrangement made it easy for the mice to reach and eat the food, but difficult for them to scatter more than a small amount. Contamination of the food in the jars was rarely seen and was always trivial in extent. Any food which was scattered was collected on the paper on which the cage rested. The jars were filled each day at the same time and the food remaining in them was weighed. The papers contaminated with urine, faeces and scattered food, were collected and dried in the air for 4 days, after which time the scattered food was separated from the faeces by rubbing the mixture through a 12-mesh sieve. Though tedious and rather distasteful, no real difficulty was encountered in making a complete separation. The food consumption was then obtained by difference. All the weighings of food were recorded to the nearest 0·25 g.

The mice were grouped according to weight so that in any one cage the weights varied from one another by no more than 3 g. The animals were weighed individually to the nearest 0·25 g. every other day throughout the experiment. They were allowed to become accustomed to life in a cage without shavings for one week before the experiment was started, when weighing of the food and the mice was begun and continued until the food intake was found to have become stabilized. At this point a selection was made in order to reduce the scatter among the body weights of the individuals in a cage. The extremes of weight were rejected and of the remaining animals exchanges were made between the cages if this would increase the uniformity. Implantation was done 2 days later.

Two sarcomas were used, Sarcoma 37 and Sarcoma 180. Sarcoma 37 was originally obtained from the Chester Beatty Research Institute of the Royal Cancer Hospital, and has been perpetuated in this laboratory for several years by serial implantation. Sarcoma 180 was obtained from the Sloan-Kettering Institute of New York through the courtesy of Dr. C. Chester Stock. This tumour was descended from the original Crocker Sarcoma 180. It was obtained from the United States after a series of ambiguous experiments with Sarcoma 37 had raised the possibility that this latter tumour might be resistant to the action of amethopterin.

Implantation of the Sarcoma 180 was done by the trocar-cannula method. Sarcoma 37 was implanted aseptically from a micrometer syringe by the method described elsewhere (Bennette, 1952). In every case 0·04 ml. of tumour mince suspension in 0·9 per cent NaCl was injected from a No. 14 needle subcutaneously into the right flank. In any one experiment all the mice were implanted with the same homogeneous tumour mince prepared by the method of Craigie (1949*a*) in a pressure mincer fitted with two plungers with groove depths of approximately 0·5 and 0·125 mm. In Experiments 1–4, a 50 per cent (v/v) mince suspension in saline was used.

Treatment was started 6 days after implantation in Experiments 1 and 2, and 10 days after implantation in Experiments 3 and 4. The sample of amethopterin used was obtained from Lederle Laboratories through the courtesy of Dr. T. H. Jukes. It was of tested purity. In all the experiments reported, solutions containing 12 mg. per cent (w/v) of amethopterin were made up freshly each day in sterile 0·1M $NaH_2PO_4$ : $Na_2HPO_4$ buffer at pH 7·8 and were stored at 0° C. Control animals received the same phosphate buffer alone. The volumes of the doses given, representing about 0·3 per cent of the body weight of the mice, were varied according to the mean weights of the groups so that the dose per kg. should be as nearly as possible the same for two groups of different mean body weight. At a site removed from the place of implantation, subcutaneous injections were given at 8-hourly intervals in accordance with the findings of the toxicity experiments.

At the end of the experiments surviving mice were killed and the tumours dissected out and examined for areas of necrosis, the degree of adherence to surrounding structures, the presence of ulceration and the vascularity. The growths were weighed wet to the nearest 10 mg. Systematic histological examination was not done in this series of experiments.

In Experiment 5, the procedure was identical to that described above except in the following respects. At the time of implantation, half the number of mice in each cage were distinguished from the rest so as to provide a total of 16 groups.

Control and experimental sets for each sex were implanted with the standard volume of tumour mince suspensions at the following dilutions (v/v) : $10^{-1 \cdot 0}$ : $10^{-1 \cdot 5}$ $10^{-2 \cdot 0}$ : $10^{-2 \cdot 5}$. Treatment was started 2 days after implantation. The appearance and development of progressively growing tumours were recorded for 19 days after implantation, during which time food intake and body weight records were made. When the mean body weights of the experimental groups showed that recovery from the effects of treatment was well established, food intake and body weight records were discontinued and the mice bearing tumours were killed. Those mice with no palpable tumours were kept under observation for another 23 days, after which it was assumed that no further tumours would appear and the animals were killed. The tumours were not weighed or subjected to post-mortem examination since it was the appearance of the growths and not the character of their subsequent development which was important in this experiment. The mice which died as a result of treatment were examined for the presence of tumours. Those which bore tumours were included in the analysis while those which showed no sign of a tumour were excluded.

*Toxicity.*

Details of the toxicity experiments are not presented. The findings of Ferguson, Thiersch and Philips (1950) were repeated. It was found that the tolerance was markedly reduced when the total dose was progressively fractionated. Thus, when the drug was given daily, the total dose tolerated was more than double that producing an equivalent condition of intoxication when the injections were given 8-hourly. The individual variation in the response of albino mice was considerable when the drug was given once daily, even in groups carefully selected for weight uniformity. This variation was substantially reduced by giving the injections three times a day. Reports of the rapid excretion of pteroylglutamic acid analogues quoted by Burchenal *et al.* (1951) and the findings of Swenseid, Swanson, Miller and Bethell (1950) support the view that the maintenance of an effective blood level would be favoured by reducing the intervals between injections. The subcutaneous route was chosen for the same reason.

### CALCULATION AND INTERPRETATION OF RESULTS.

The mice comprising some of the control and all of the experimental groups were housed in more than one cage, and since it was necessary to make selections in order to reduce the scatter among the individual body weights of the animals, it was not possible to regard the groups as constituting a single population. The body weight curves were accordingly calculated for each cage separately and then t-tested one against the other. In most cases no significant difference was found and grouping was therefore possible. In the following sets however—Experiment 2 female experimental : Experiment 4 male experimental : Experiment 5, male control—small but significant differences were found between the body weight curves derived from different cages. In each case the displaced curves pursued courses roughly parallel to the others up to the time of treatment, and it was considered likely that a simple procedure for making a correction for the displacement could be justified. The mean difference between corresponding points on the curves was obtained and the assumption that this value represented the extent of a simple displacement of the origin was tested by repeating the t-test on the pairs of points obtained by appropriately adding or subtracting the mean difference. No significant difference was found in any of the instances after the correction had been made and it was considered unnecessary to do a full analysis of covariance. Having obtained single curves for the grouped control and grouped experimental sets for each experiment, these were now tested for significant difference up to the onset of treatment. In Experiment 3 (females) and Experiment 5 (males and females) significant displacements were found to exist. These were corrected for as has been described, the mean difference being added in each case to the curve with the lower origin. As before the procedure was justified by the finding that no significant differences existed after the corrections had been made. The estimated terminal mean body weight values were corrected for the presence of the tumours by subtracting the estimated mean tumour weights from the terminal mean body weight values, and the body weight changes were expressed as percentage reductions on the mean control values. Since the control tumours never reached such a size as to cause any obvious constitutional disturbance in the mice, it could be assumed fairly confidently that their presence had a comparatively small effect on the body weight. The error due to the difference in tumour sizes between control and experimental animals, if their presence were responsible for a loss in body weight, would result in an understatement in the percentage weight change obtained. The temporary depression or flattening of the ascending weight curves of the control groups, corresponding to the period of injections, may be taken to be due to the disturbance produced by the extra handling involved. The body weight figures refer only to those mice which survived the experiments, including those described as moribund at the end. This has been done in order to exclude the " effect of death " on the body weight, a sudden profound terminal loss due to dehydration. In a few cases the moribund mice may have been in this state, but the easy access to water would mean that mice in an advanced toxic state would have to be virtually immobilized before terminal dehydration could set in. Mice referred to as " moribund " were those within, say, 48 hours of death—ruffled, unsteady and suffering in most cases from diarrhoea which was haemorrhagic in some instances. A distinction is drawn between the use of the words " moribund " and " dying." It was found in most cases that a ruffled, unsteady mouse described as moribund would pass into an agonal state in which it would become grossly ataxic and finally cease to be able to move about the cage. The rate at which this sequence of events occurred was rather variable, and in order to account for this, an arbitrary correction was made by excluding dying mice from the numbers of the groups on the day before their death when calculating the food intake per mouse for that day. The food intake figures were obtained from data relating to the whole group. The mean food intake per mouse was calculated for each cage separately over 4-day periods, and the results were subjected to the same analysis as that applied to the body weight results. The procedure proved to be satisfactory in every case but one (Experiment 3, females). In this group there were significant differences before, during and after treatment, and these were not consistent. The result has been recorded as of doubtful significance.

The quantitative estimates set down in Table I were derived from data for which the values of the significance P were in every case less than 0·01. A less exacting criterion of significance is regarded as being inappropriate in work of this kind.

TABLE I.—*Showing Relation Between Toxicity and Tumour Inhibition Due to Amethopterin.*

| Experiment No. and description of group. | | | Number of injections. | Total dose mg. per kilo. | Body wt. loss $\frac{\text{con.-exp.}}{\text{con.}}\times 100.$ | Food intake reduction $\frac{\text{con.-exp.}}{\text{con.}}\times 100.$ | Number of deaths. | Number terminally moribund. | Tumour wt. loss $\frac{\text{con.-exp.}}{\text{con.}}\times 100.$ | Total take rate. |
|---|---|---|---|---|---|---|---|---|---|---|
| 1 S180 | ♀ | Con. | 10 | — | (0·05 <P <0·1) | 12·9 ± 2·6 | 0/14 | 0 | (0·02 <P <0·05) | 88/90 |
| | | Exp. | | 4·0 | | | 1/30 | 0 | | |
| | ♂ | Con. | 10 | — | 13·3 ± 3·4 | 42·4 ± 5·5 | 0/15 | 0 | . 38·0 ± 10·0 | |
| | | Exp. | | 4·0 | | | 5/29 | 12 | | |
| 2 S 180 | ♀ | Con. | 13 | — | 17·5 ± 2·0 | 45·2 ± 5·3 | 0/15 | 0 | . 58·5 ± 8·5 | 85/90 |
| | | Exp. | | 5·2 | | | 7/27 | 9 | | |
| | ♂ | Con. | 8 | — | (0·1±P±0·2) | (0·1 <P <0·2) | 0/15 | 0 | (0·2 <P <0·3) | |
| | | Exp. | | 3·2 | | | 2/28 | 0 | | |
| 3 S 37 | ♀ | Con. | 10 | — | (0·1 <P <0·2) | (Doubtful significance) | 0/11 | 0 | (0·2 <P <0·3) | 103/105 |
| | | Exp. | | 4·0 | | | 0/20 | 0 | | |
| | ♂ | Con. | 10 | — | 13·6 ± 1·7 | 34·2 ± 5·2 | 0/10 | 0 | . 38·5 ± 8·0 | |
| | | Exp. | | 4·0 | | | 2/22 | 14 | | |
| 4 S37 | ♀ | Con. | 13 | — | 16·9 ± 1·8 | 54·8 ± 5·6 | 0/10 | 0 | . 57·1 ± 9·5 | 119/120 |
| | | Exp. | | 5·2 | | | 11/33 | 13 | | |
| | ♂ | Con. | 8 | — | (0·4 <P <0·5) | 9·8 ± 3·1 | 0/9 | 0 | (0·05 <P <0·1) | |
| | | Exp. | | 3·2 | | | 1/29 | 0 | | |
| 5 S37 | ♀ | Con. | 13 | — | 23·5 ± 1·3 | 61·8 ± 4·2 | 0/37 | $TPD_{50}$ calculated from number of takes at mince dilutions $10^{-1.0}:10^{-1.5}:10^{-2.0}:10^{-2.5}$ | | $10^{-1.95}\pm 0.22$ |
| | | Exp. | | 5·2 | | | 13/37 | | | $10^{-1.66}\pm 0.23$ |
| | ♂ | Con. | 9 | — | 11·2 ± 2·3 | 52·4 ± 4·1 | 0/36 | | | $10^{-1.89}\pm 0.22$ |
| | | Exp. | | 3·6 | | | 8/40 | | | $10^{-1.79}\pm 0.24$ |

$\frac{\text{Con.-Exp.}}{\text{Con.}}\times 100$ signifies the ratio of the difference between the estimated mean values for the control and experimental groups to the estimated mean control value, expressed as a percentage. Quantitative estimates have been given under this heading only in cases where the control and experimental values differed significantly, taking P = 0·01 as the criterion of significance.

$TPD_{50}$ represents the estimated dilution of tumour mince which would have produced tumours in 50 per cent of the mice.

Fig. 1 and 2 show the food intake and body weight curves in Experiments 3 and 2 respectively. The corresponding curves for Experiments 1 and 4 are essentially the same. Since all the corresponding points have been t-tested, the

FIG. 1.—Experiment 3. Sarcoma 37. Body weight and food intake. - - - - - - Treated group. ——— Control group.

individual values for the standard errors about the means have been omitted from the figures for the sake of clarity.

In Experiments 1 and 3, the males and females received exactly the same treatment and it is seen that it was only in the males that any important degree of intoxication occurred and that tumour growth retardation likewise only occurred to a significant extent in these groups. In Experiments 2 and 4 the does

FIG. 2.—Experiment 2. Sarcoma 180. Body weight and food intake. - - - - - - Treated group. ——— Control group.

given to the females was increased and that given to the males decreased, with the result that the females exhibited the manifestations of toxicity and also showed a retardation of the growth of the tumours they carried. The males showed no such changes to any important extent. For the males, a 20 per cent reduction in the total dose producing intoxication and tumour growth retardation failed to produce either to a significant degree. For the females the corresponding figure was 23 per cent.

Micrococci and beta-haemolytic streptococci were found to have contaminated the minces used for implanting the tumours in Experiments 3, 4 and 5, in spite of aseptic precautions. This problem was encountered by Warner and Gostling (1950) and in common with these workers it is felt that while it is important to check the degree of contamination, the presence of bacteria does not necessarily invalidate the results when experiments are adequately controlled.

No obviously consistent differences were found between the naked eye appearances of control and experimental tumours and the records have not been analysed in detail.

In Experiment 5, the 50 per cent end point of the " titration ' has been calculated by Thompson's method (1947), this being recommended by Armitage and Allen (1950) as a reliable short-cut method in which the result can be expressed within 95 per cent fiducial limits. The index used by Warner and Gostling (1950) has not been calculated. Since the standard error of the difference between the "tumour producing dose $_{50}$" ($TPD_{50}$) for the control and experimental sets is greater than the difference itself, the shifts in the end points as a result of treatment at a toxic level of dosage cannot be regarded as significant.

### DISCUSSION.

The results of Experiments 1 to 4 show that retardation of the tumours Sarcoma 180 and Sarcoma 37 only occurs to an important extent when the hosts are intoxicated by amethopterin to the point where about half the animals have either died or passed into an advanced toxic state as a result of treatment under the conditions stated. The question to be decided is the extent to which the action of the drug can be regarded as selective in its effect upon the tumours. Lees and Lees (1950) repeatedly found that non-specific toxic substances with low therapeutic ratios would bring about " significant " retardations of tumour growth. One main purpose of this report is to submit an additional emphasis to the recommendation made by these workers that any drug with a low therapeutic ratio may be responsible for unjustifiable optimism as to its tumour retarding activity if the basic considerations of the therapeutic ratio are not fully taken into account. Rous (1914), Bischoff, Long and Maxwell (1935), Bischoff and Long (1938), Tannenbaum (1947) and Goldin and his associates (1949) are among those who have shown that the growth of recent implants is markedly affected by restriction of the caloric intake. The problem of deciding between selective and unselective toxicity has been approached by Goldin and his co-workers (1949) in experiments with nitrogen mustards. These workers have studied the effect of dietary restriction on the growth of Sarcoma 180 and concluded from paired feeding experiments that the weight loss incurred by the treated animals could account for the whole of the tumour growth retardation observed in every case but one of a series of toxic nitrogen mustards. By reference to a regression line relating the percentage tumour weight and the percentage body weight loss, it was stated that only 29 per cent of the total tumour regression due to this one compound could be attributed to a specific effect of the drug.

The use of a regression line as a means of assessing the utility of a toxic chemotherapeutic agent may be useful as a means of excluding compounds the toxicity of which is such that the body weight loss caused by their use can be shown to be sufficient to bring about the whole of the tumour growth retardation observed. But where a retardation occurs which is greater than can be accounted for in this way, the assertion that this residual activity is specific implies that body weight loss is taken to be a completely satisfactory criterion of chronic toxicity. Paired feeding experiments designed to establish regression lines for the tumours used in the work reported here were not done because it is held that no single criterion is adequate for the quantitative expression of chronic poisoning. Furthermore, it cannot be assumed that information derived from calorie restriction experiments can be applied directly to cases where the reduced food intake is the result of anorexia due to slow poisoning, with all the complex metabolic disorder which must occur in such a state. The use of a regression line as a means of making a quantitative correction for the effect of calorie restriction on the growth of a tumour is felt to be of little use if it remains impossible to have confidence that the residual activity really represents a specific action on the part of the drug. If the effects of intoxication on the growth of a tumour cannot be separated from a supposedly selective effect of the drug causing the intoxication, it must also be impossible to apply the conventional approach of chemotherapy to the study of animals bearing tumours—that is, the attempt to enhance the specific action and reduce the toxicity by correlating the biological activity with the chemical structure. On theoretical grounds, then, it is submitted that the study of the effect of drugs on the growth of tumours in animals is not profitable when the compounds are active only at lethal or almost lethal levels. Such a conclusion is by no means original, but no apology is offered for its restatement because it is clear that much careful work continues to be done without full recognition of the fact that the results in many instances cannot be interpreted unequivocally by reason of these considerations.

The titration method used by Warner and Gostling (1950) was adopted as a means of investigating the effect of amethopterin on the tumour producing potency of a mince of Sarcoma 37. Exposure of a dispersed implant to amethopterin at a toxic level shortly after implantation was thought likely to represent a condition in which the tumour cells would have the maximum susceptibility to damage. The fact that no significant alteration in the estimated 50 per cent end point was discovered is surprising in view of the findings of Tannenbaum (1947) relating to the effect of calorie restriction on the early development of tumours. But the failure by this method to demonstrate an action of the drug on the tumour cells is not surprising in view of the failures reported in attempts to show a specific action *in vitro* (Stock, Biesele, Burchenal, Karnofsky, Moore and Sugiura, 1950). The titration method has the advantage of overcoming many of the difficulties inherent in the quantitative evaluation of tumour growth in terms of growth rate. Its applicability to experimental cancer chemotherapy is being investigated. The criticism of the method advanced by Craigie (1949*b*) has been noted. It would appear that the objection applies more particularly to cases where comparisons are attempted between experiments in which difference minces have been prepared.

The negative result of Experiment 5 taken in conjunction with the fact that a reduction by one fifth of a toxic and tumour retarding dose produces neither tumour inhibition nor signs of toxicity under the conditions of the experiments is taken to indicate that the association between toxicity and tumour inhibition is too close to make it possible to place any reliance on the selectivity of the tumour retarding effect of amethopterin for the transplanted Sarcomas 37 and

180. The difference between the conclusion reached here and that given by other workers in this field, notably Stock and his associates (1950), seems likely to be due essentially to the fact that the use of an 8-hourly treatment procedure made it possible to work in the neighbourhood of the extreme limit of tolerance to the drug and to define more narrowly the conditions prevailing at the toxic and just subtoxic levels of dosage. The possibility cannot be overlooked that tumour tissue might be selectively damaged when exposed to higher concentrations of amethopterin intermittently while it is resistant to a more continuous exposure to lower concentrations, such as would be produced by more frequent injections.

### SUMMARY.

Experiments have been described in which the tumour retarding action of 4-amino-$N_{10}$ methyl pteroylglutamic acid (amethopterin) in mice has been related to the toxic state induced in the animals as a result of treatment. The degree of chronic intoxication has been judged by the food intake and body weight changes which occur. The conclusion has been reached that the anti-neoplastic activity of the drug is so closely associated with manifestations of toxicity that no confidence can be placed in the selectivity of its action against the two transplanted sarcomas studied. The sex difference in toxicity has been confirmed and the failure to obtain a significant retardation of tumour growth except at the toxic levels of dosage for the two sexes supports the conclusion that the retardation is, to a great extent at least, the result of an unselective poisoning. Some of the problems connected with the evaluation of toxic chemotherapeutic agents in this field have been discussed.

The support of the British Empire Cancer Campaign, which has borne the expense of this work, is gratefully acknowledged. It is a pleasure also to extend thanks to Professor F. Dickens and Professor E. C. Dodds for their help and encouragement, to Dr. C. T. Beer for help during part of the experimental work, and to Dr. P. Armitage of the Medical Research Council's Statistical Research Unit for his advice in connection with the statistical analysis.

### REFERENCES.

Armitage, P., and Allen, Irene.—(1950) *J. Hyg., Camb.*, **48**, 298.
Bennette, J. G.—(1952) *Brit. J. Cancer*, **6**, 389.
Bischoff, F., and Long, M. L.—(1938) *Amer. J. Cancer*, **32**, 418.
*Iidem* and Maxwell, L. C.—(1935) *Ibid.*, **24**, 549.
Burchenal, J. H., Karnofsky, D. A., Kingsley-Pillers, Elizabeth, M., Southam, C. M., Laird Myers, W. P., Escher, G. C., Craver, L. F., Dargeon, H. W., and Rhoads, C. P.—(1951) *Cancer*, **4**, 549.
Craigie, J.—(1949*a*) *Brit. J. Cancer*, **3**, 249.—(1949*b*) *Brit. Med. J.*, ii, 1485.
Farber, S., Diamond, L. K., Mercer, R. D., Sylvester, R. F., jr., and Wolff, J. A.—(1948) *New Engl. J. Med.*, **238**, 787.
Ferguson, F. C., jun., Thiersch, J. B., and Philips, F. S.—(1950) *J. Pharmacol.*, **98**, 293.
Goldin, A., Goldberg, B., Ortega, L. G., Fugmann, Ruth, Faiman, Frieda, and Schoenbach, E. B.—(1949) *Cancer*, **2**, 865.
*Idem*, Greenspan, E. M., Goldberg, B., and Schoenbach, E. B.—(1950) *Ibid.*, **3**, 849.
Lees, T. W., and Lees, J. C.—(1950) *Ibid.*, **3**, 580.

MOORE, ALICE E., STOCK, C. C., SUGIURA, K., AND RHOADS, C. P.—(1949) *Proc. Soc. exper .Biol., N.Y.*, **70**, 396.
PETERING, H. G.—(1952) *Physiol. Rev.*, **32**, 197.
ROUS, P.—(1914) *J. Exp. Med.*, **20**, 433.
SCHOENBACH, E. B., GOLDIN, A., GOLDBERG, B., AND ORTEGA, L. G.—(1949) *Cancer*, **2**, 57.
STOCK, C. C., BIESELE, J. J., BURCHENAL, J. H., KARNOFSKY, D. A., MOORE, ALICE, E., AND SUGIURA, K.—(1950) *Ann. N.Y. Acad. Sci.*, **52**, 1360.
SUGIURA, K., MOORE, ALICE E., AND STOCK, C. C.—(1949) *Cancer*, **2**, 491.
SWENSEID, M. E., SWANSON, A. L., MILLER, S., AND BETHELL, F. H.—(1950) *Fed. Proc.*, **9**, 372.
TANNENBAUM, A.—(1947) "The Role of Nutrition in the Origin and Growth of Tumours." (A.A.A.S.) 'Approaches to Tumour Chemotherapy.' pp. 96–127.
THOMPSON, W. R.—(1947) *Bact. Rev.*, **11**, 115.
WARNER, P. T. J. C. P., AND GOSTLING, J. V. T.—(1950) *Brit. J. Cancer*, **4**, 380.


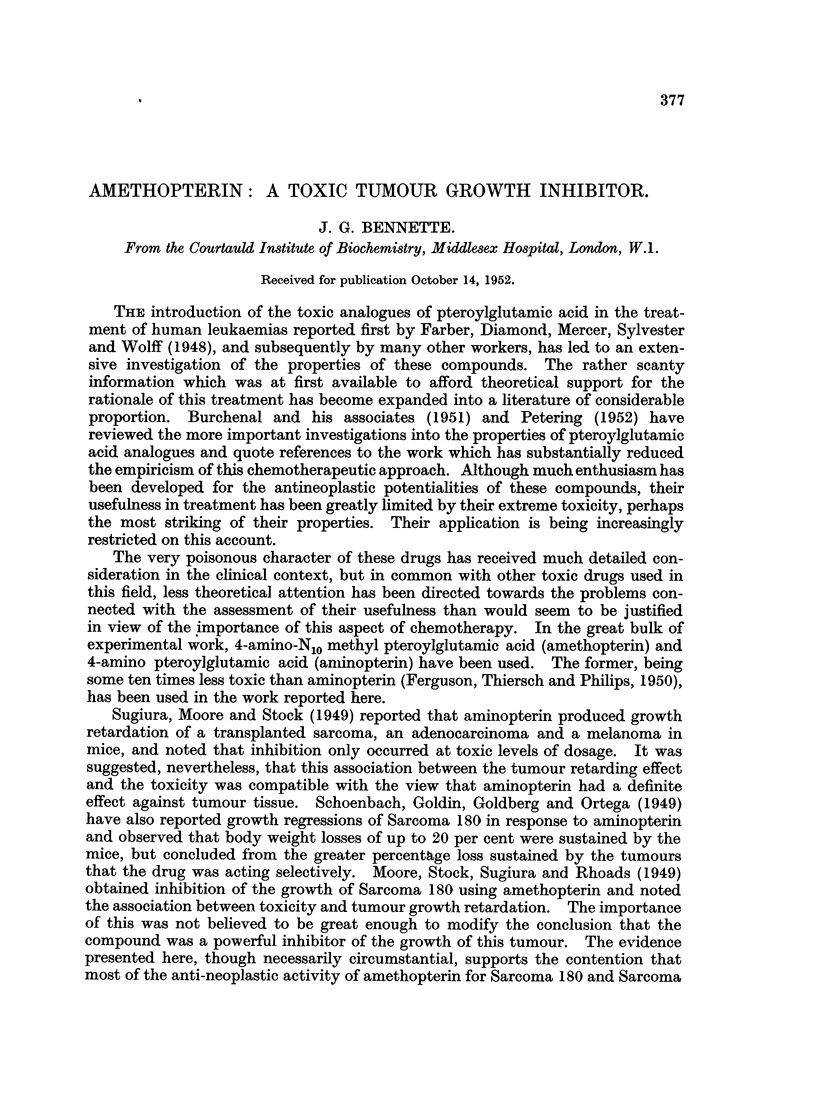

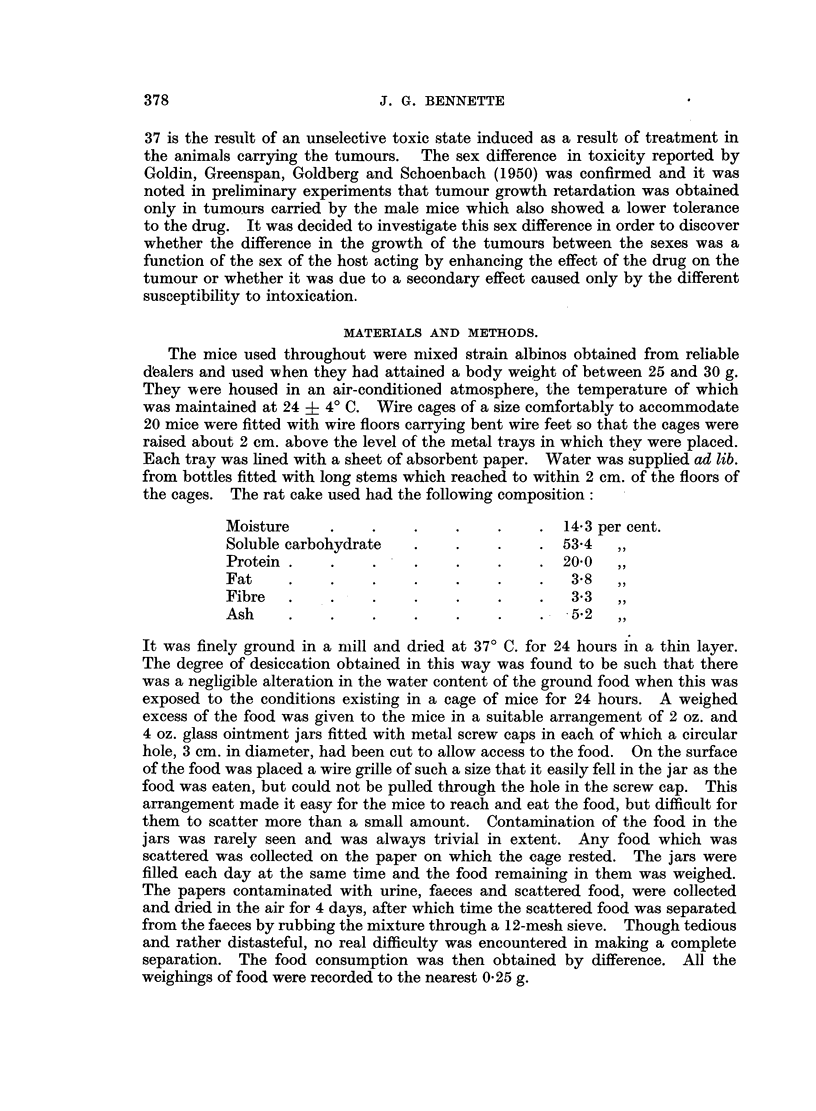

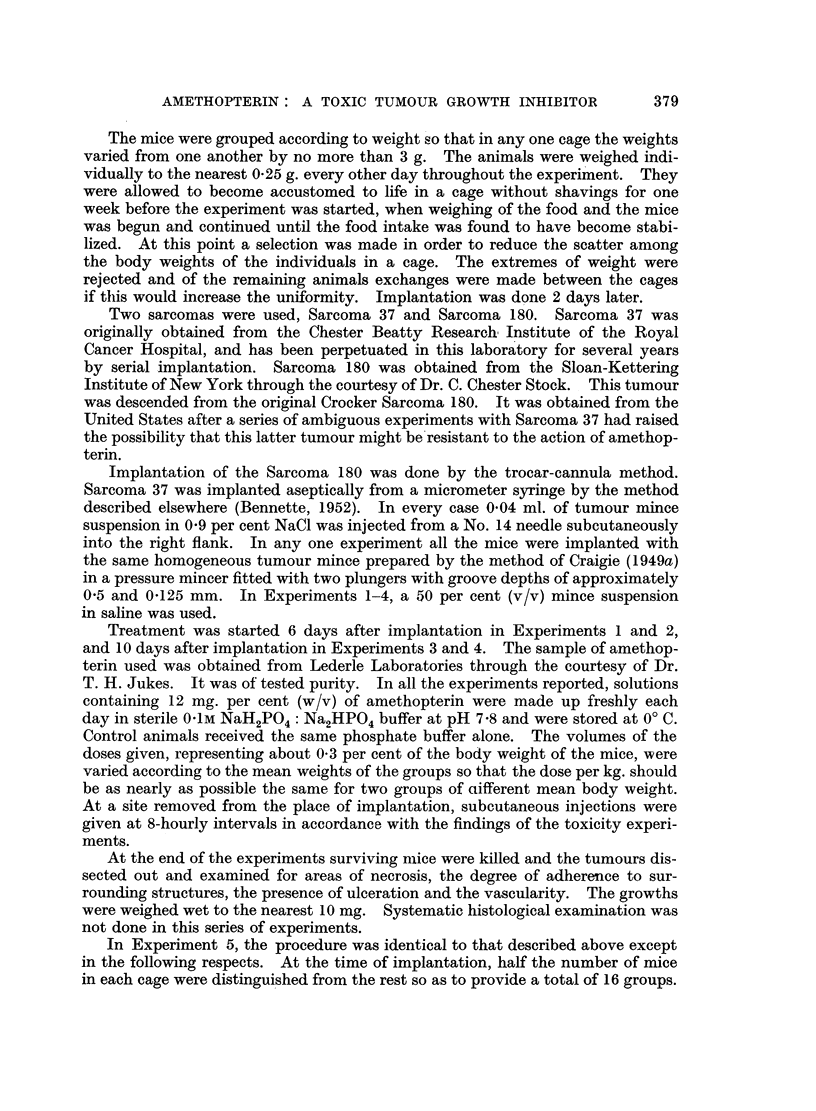

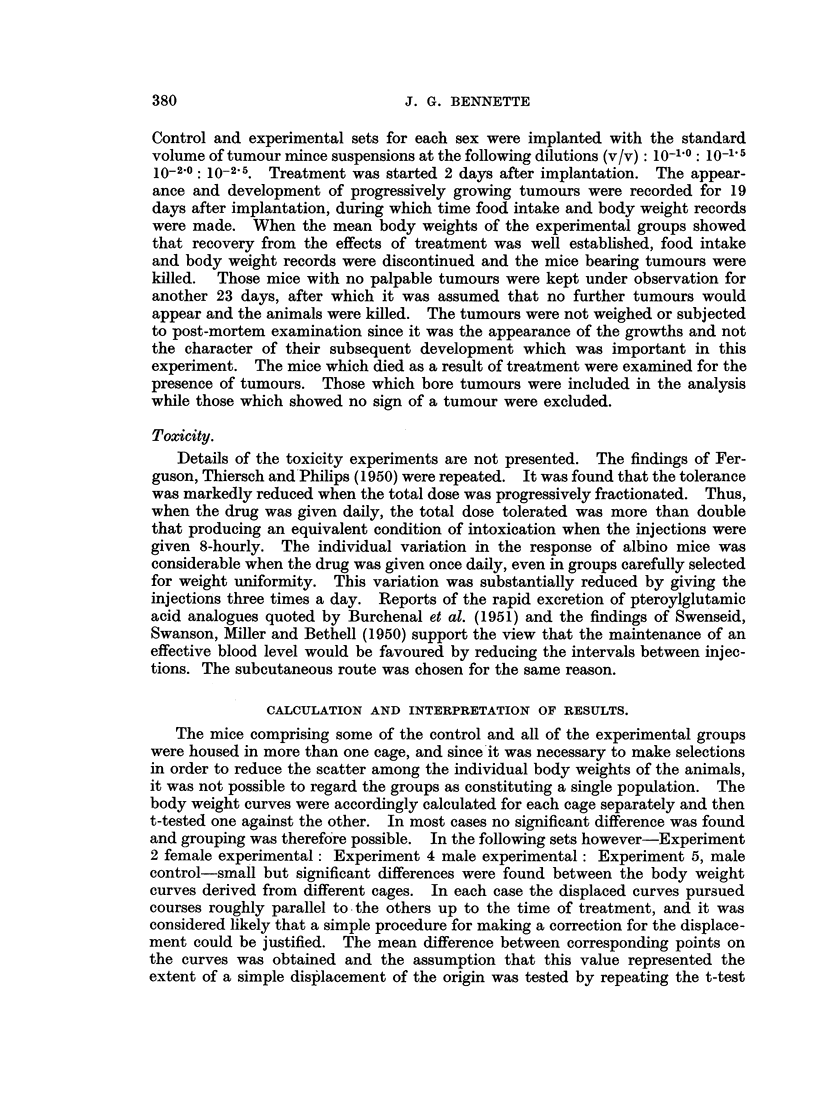

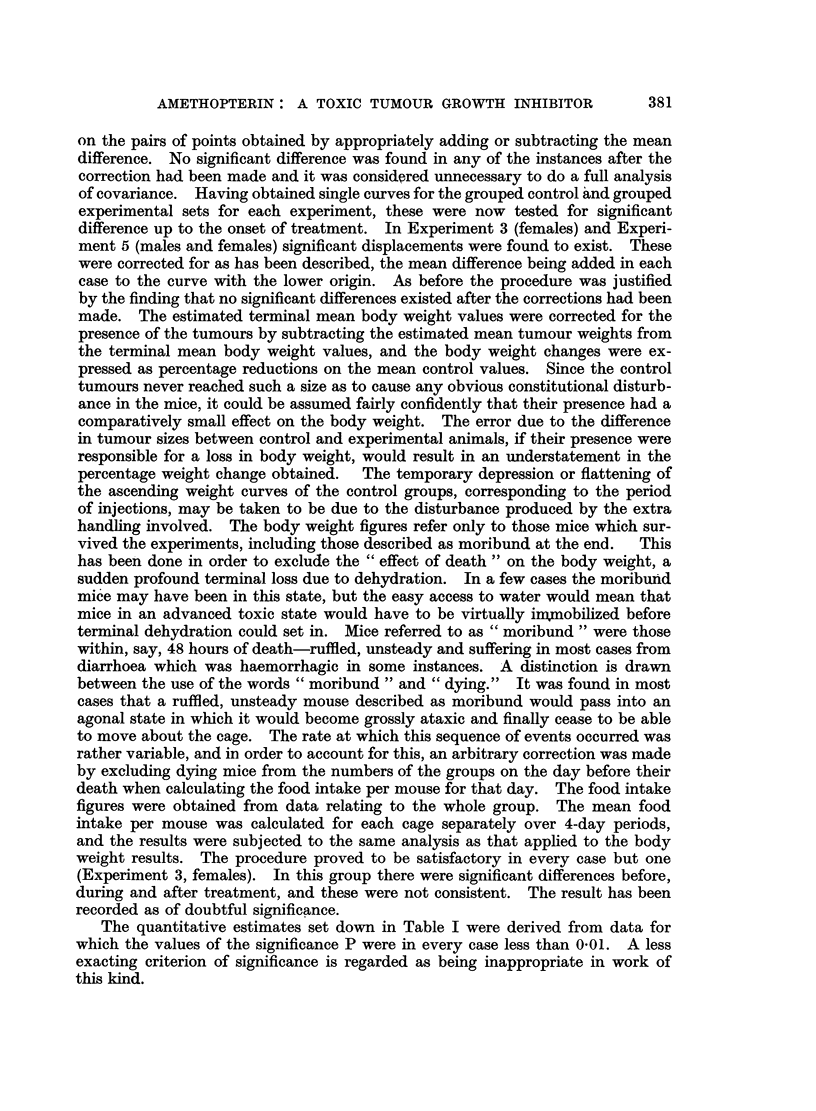

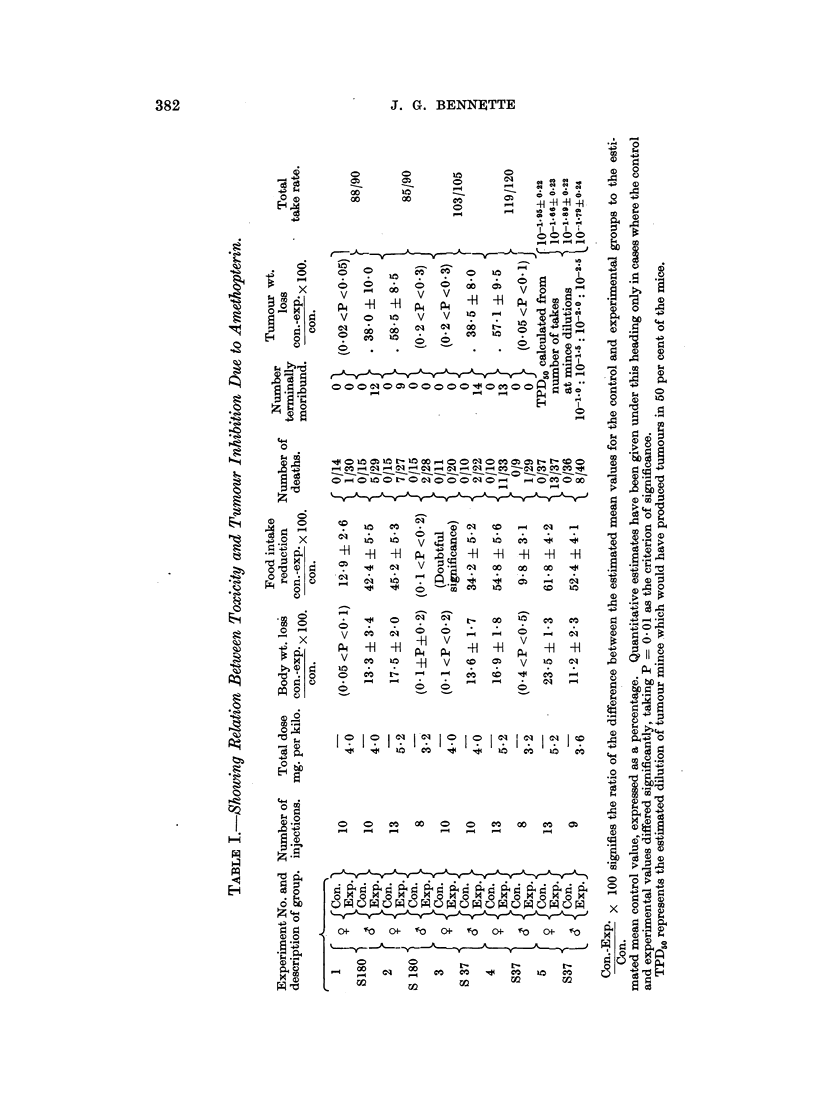

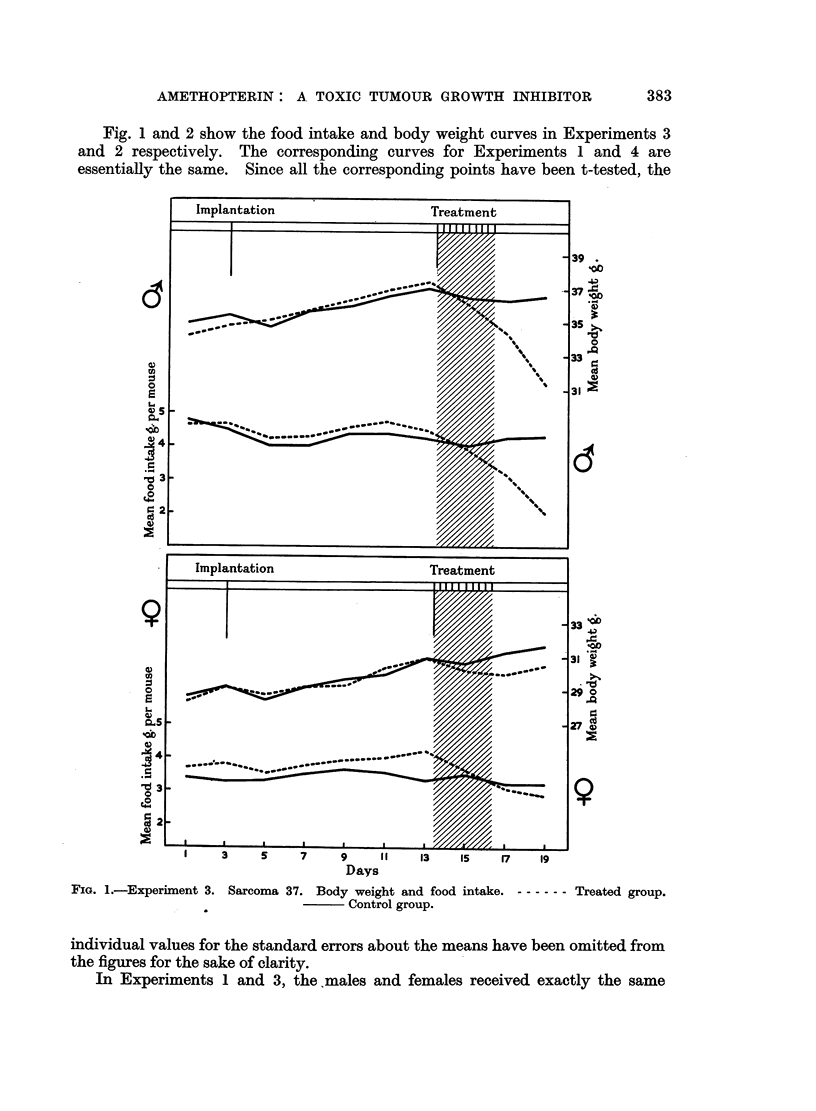

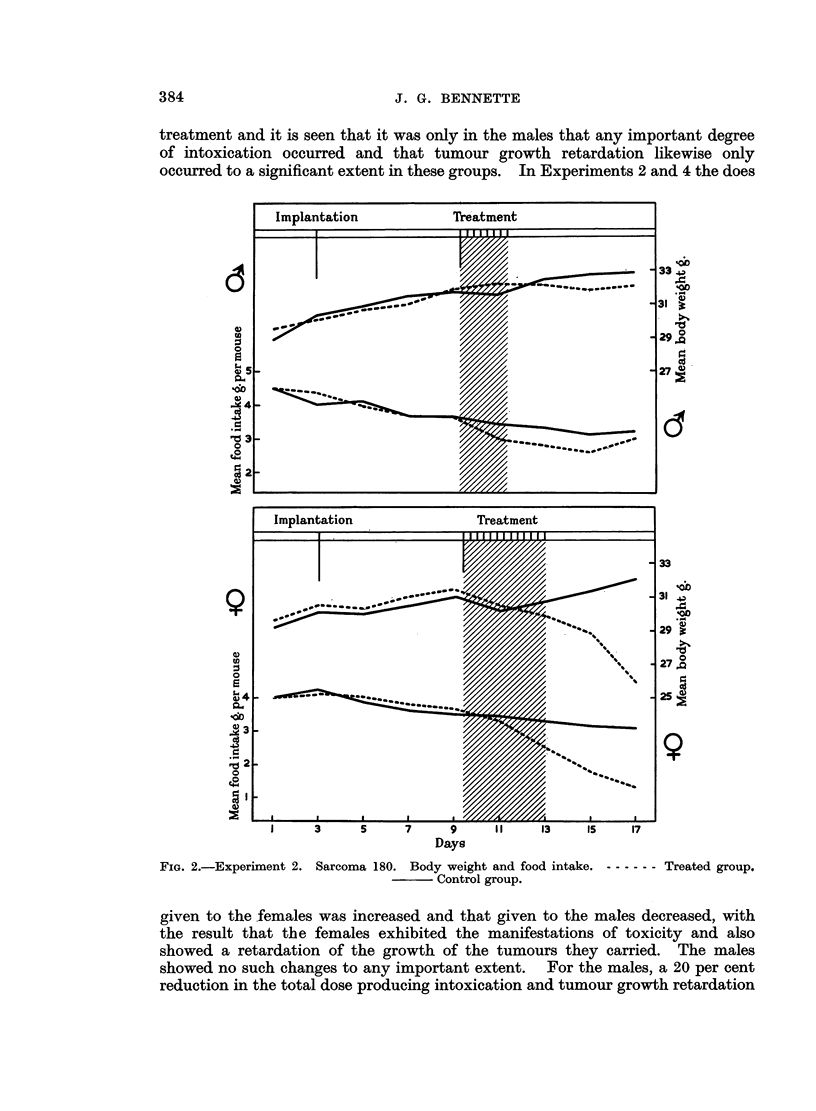

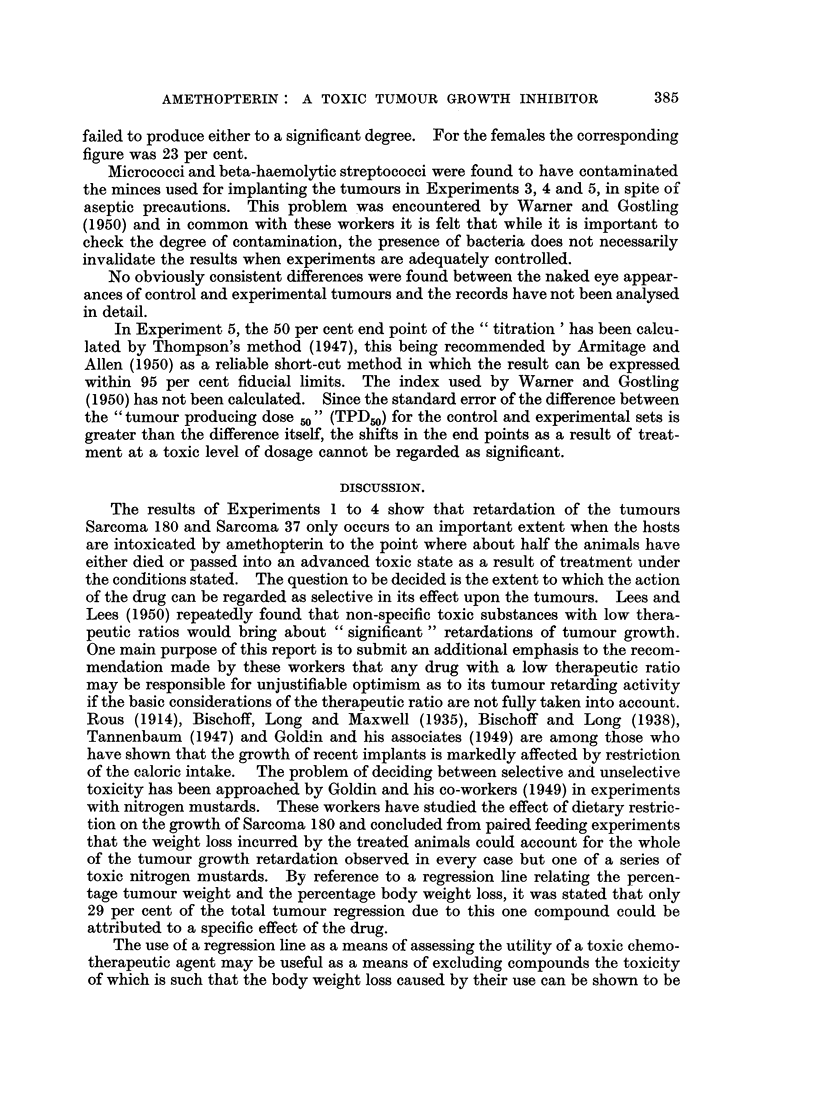

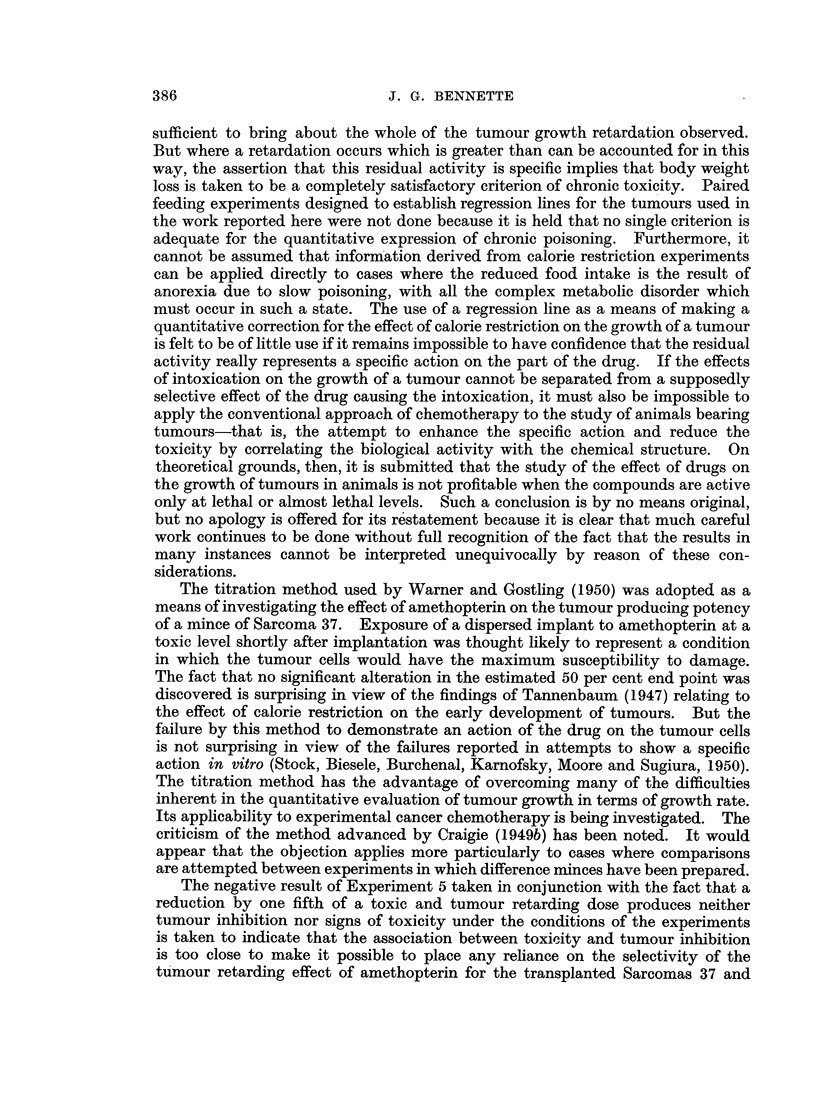

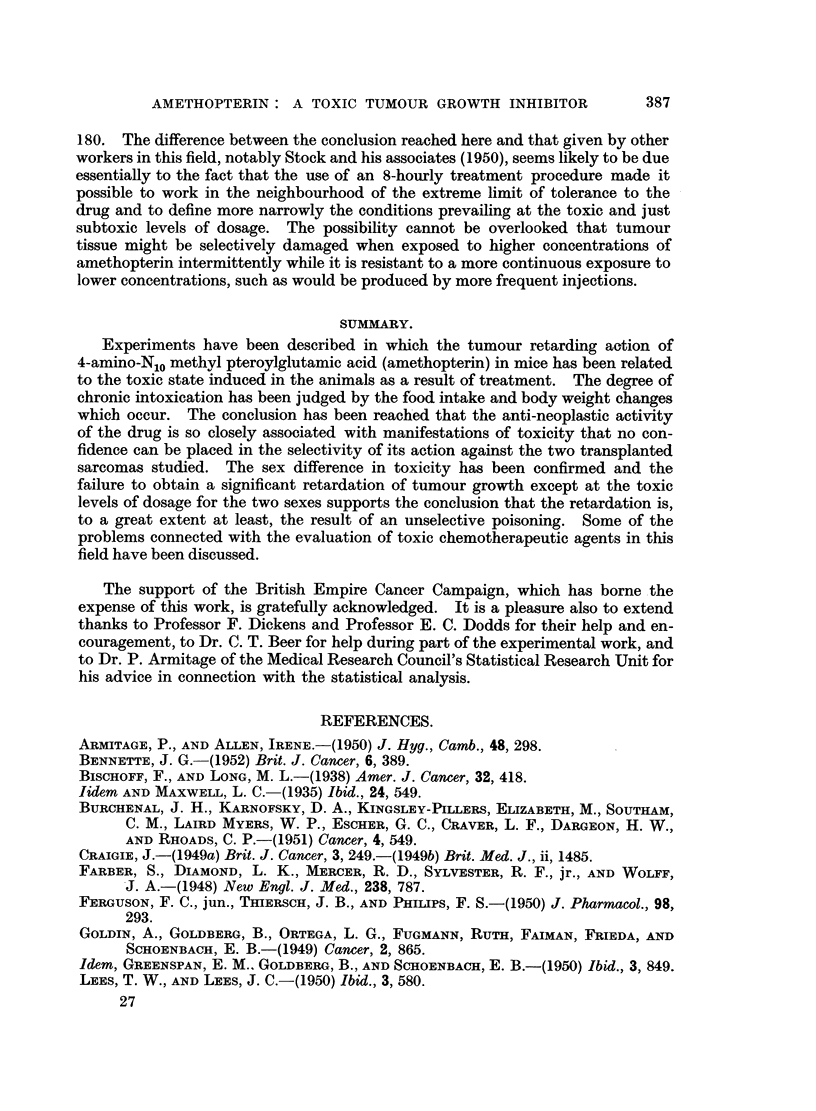

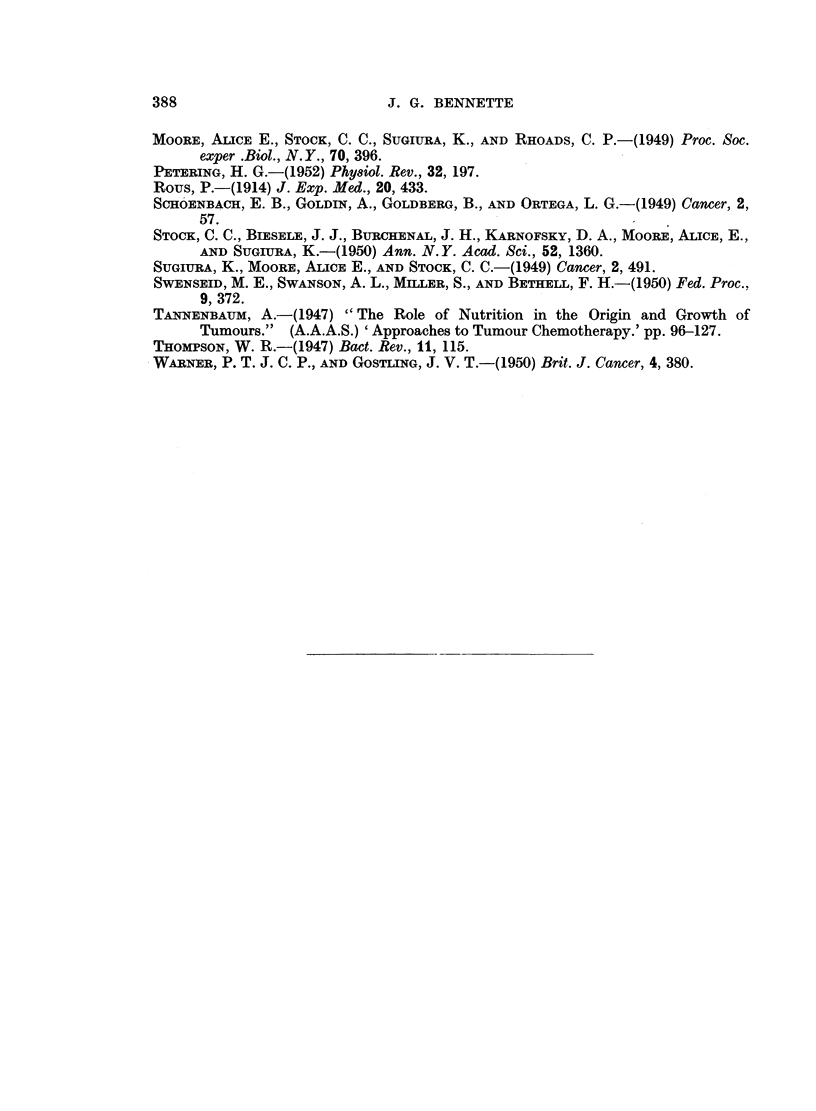


## References

[OCR_00579] BENNETTE J. B. (1952). A standard procedure for implanting tumour cell suspensions.. Br J Cancer.

[OCR_00587] BURCHENAL J. H., KARNOFSKY D. A., KINGSLEY-PILLERS E. M., SOUTHAM C. M., MYERS W. P. L., ESCHER G. C., CRAVER L. F., DARGEON H. W., RHOADS C. P. (1951). The effects of the folic acid antagonists and 2,6-diaminopurine on neoplastic disease, with special reference to acute leukemia.. Cancer.

[OCR_00601] GOLDIN A., GREENSPAN E. M., GOLDBERG B., SCHOENBACH E. B. (1950). Studies on the mechanism of action of chemotherapeutic agents in cancer. I. A sex difference in toxicity to the folic acid analogue, 4-amino-pteroylglutamic acid.. Cancer.

[OCR_00615] PETERING H. G. (1952). Folic acid antagonists.. Physiol Rev.

[OCR_00623] STOCK C. C., BIESELE J. J., BURCHENAL J. H., KARNOFSKY D. A., MOORE A. E., SUGIURA K. (1950). Folic acid analogs and experimental tumors.. Ann N Y Acad Sci.

[OCR_00636] WARNER P. T. J. C. P., GOSTLING J. V. T. (1950). The effect of freezing and freeze-drying on the transplantation of sarcoma 37.. Br J Cancer.

